# Radioresistant triple-negative breast cancer cells release β-catenin containing extracellular vesicles to promote cancer stem cell activity of bystanders

**DOI:** 10.7150/jca.111555

**Published:** 2025-06-23

**Authors:** Yueh-Chun Lee, Peng-Ju Chien, Yu-Ting Chang, Yu-Hao Huang, Chin-Fang Chang, Shao-Ti Li, Wen-Wei Chang

**Affiliations:** 1 Department of Radiation Oncology, Chung Shan Medical University Hospital, Taichung, Taiwan.; 2 School of Medicine, Chung Shan Medical University, Taichung, Taiwan.; 3 Department of Biomedical Sciences, Chung Shan Medical University, Taichung, Taiwan.; 4 Rayon Clinic, Taichung, Taiwan.; 5 Department of Otorhinolaryngology, Head and Neck Surgery, Taichung Hospital Ministry of Health and Welfare, Taichung, Taiwan.; 6 Rong Hsing Research Center for Translational Medicine, National Chung Hsing University, Taichung, Taiwan.; 7 Department of Medical Research, Chung Shan Medical University Hospital, Taichung, Taiwan.

**Keywords:** triple negative breast cancer, extracellular vesicles, radioresistance, β-catenin, cancer stem cells

## Abstract

**Background:** Triple-negative breast cancer (TNBC) frequently develops radioresistance, yet the mechanisms remain incompletely elucidated. This study is the first to investigate how β-catenin, transported by extracellular vesicles (EVs) from radioresistant TNBC cells, promotes radioresistance and enhances cancer stem cell (CSC) activity in recipient TNBC cells, offering a novel mechanism distinct from prior EV-related findings in other cancers.

**Methods and Results:** A radioresistant cell line (231-RR) was developed from MDA-MB-231 cells, and EVs were isolated for characterization. EVs from 231-RR cells decreased radiosensitivity in parental MDA-MB-231 and two other TNBC cell lines (MDA-MB-468 and Hs578T), as shown by clonogenic assay. These EVs also enhanced CSC activity in MDA-MB-231 and Hs578T cells, demonstrated through primary and secondary mammosphere formation. The effects were nullified when using EVs from 231-RR cells treated with the EV secretion inhibitor GW4869. 231-RR-derived EVs showed elevated β-catenin levels and increased active β-catenin and stemness proteins (c-Myc, OCT4, SOX2) in recipient TNBC cells. The β-catenin inhibitor CCT-031374 prevented EV-mediated enhancement of radioresistance and CSC activity. Public data analysis from breast cancer patients revealed post-radiotherapy upregulation of the β-catenin pathway, with elevated *CTNNB1*, *MYC*, and *CD44* expression, alongside reduced *CDKN2A* and *CDH1* levels, supporting clinical relevance.

**Conclusions:** This study uniquely demonstrates that EVs from radioresistant TNBC cells transfer β-catenin to confer radioresistance and enhance CSC activity in recipient cells, a mechanism not previously reported in TNBC. These findings suggest the potential of EV-β-catenin derived as a novel biomarker for predicting radiotherapy outcomes and recurrence risk in TNBC patients, pending development of sensitive detection methods.

## Background

Breast cancer is one of the major cancers in women worldwide. Based on the receptors on the surface of breast cancer cells, such as the hormone receptors estrogen receptor (ER) and progesterone receptor (PR) and the human epidermal growth factor receptor 2 (HER2), four subtypes can be distinguished: Luminal A (Luminal A, ER+/PR+ or -/HER2-), Luminal B (Luminal A, ER+/PR+ or -/HER2-) and Luminal B (Luminal A, ER+/PR+ or -/HER2-). HER2-positive (HER2+) and triple-negative breast cancer (TNBC, ER-/PR-/HER2-), of which TNBC is a highly malignant subtype of breast cancer 1, 2. Due to the lack of ER, PR and HER2 on the cell surface of TNBC cells, TNBC is characterized by rapid cell growth, easy recurrence, poor progression-free survival and overall survival, and susceptibility to distant metastasis 3-5. Due to the lack of suitable cell receptors, the use of targeted therapy is usually ineffective. Currently, treatment options of TNBC are mainly based on surgery combined with chemotherapy and radiotherapy 6, 7. Radiotherapy is an important tool in the fight against TNBC, but it is easy to find that patients become resistant to radiotherapy during treatment, leading to worse-than-expected outcomes and prognosis 8. Clinical data also show that patients with TNBC are more likely to relapse within a shorter time after radiotherapy than patients with other breast cancer subtypes 9. Understanding how TNBC is resistant to radiation is important for developing therapies.

Cancer stem cells (CSCs), a small subpopulation of cancer cells with stem cell-like properties, have been identified in many cancer types. These cells are thought to play crucial roles in tumor initiation, drug resistance, and metastasis 10-12. Compared to conventional cancer cells, CSCs possess enhanced DNA repair capabilities and superior regulation of oxidative stress, contributing to their increased resistance to radiation 13-15. Extracellular vesicles (EVs) are externally secreted vesicles with a phospholipid bilayer structure, containing nucleic acids, proteins, and lipids 16. In recent years, EVs have been recognized as important mediators of intercellular communication. Cancer cell-derived EVs can influence various aspects of tumor biology, including proliferation, angiogenesis, metastasis, and the tumor microenvironment 17, 18. EVs have also been implicated in cancer radioresistance. The composition of EVs can be altered when cancer cells are exposed to radiation 19, 20. For instance, in oral squamous cell carcinoma, radioresistant cells can deliver miR-503-3p via EVs to radiosensitive cells, suppressing BAK levels and reducing radiation-induced apoptosis 21. In TNBC, numerous EV-packaged miRNAs have been reported to contribute to malignant features, including the enhancement of CSC activity, and have potential applications in diagnosis 22. However, information regarding protein cargo delivery through EVs in TNBCs and its association with radiation responses remains limited.

We have previously established a radioresistant subline of MDA-MB-231 TNBC cells, called as 231-RR cells, which have high CSC activity and increased intracellular Notch1 activity 23. In this study, we investigated whether EVs derived from 231-RR cells have the ability to alter the radiation response and CSC activity of bystander cancer cells. We also investigated whether β-catenin is a key molecule involved.

## Materials and Methods

### Cell culture

Human triple-negative breast cancer (TNBC) cell lines included MDA-MB-231 (referred to as 231-P in this study), MDA-MB-468, and Hs578T cells were cultured in 6 cm dishes containing DMEM/F12 culture medium (Gibco™, Thermo Fisher Scientific, Waltham, MA, USA) supplemented with 10% fetal bovine serum (HyClone, Logan, UT, USA), 1 mM sodium pyruvate (Gibco), 2 mM L-glutamine (Gibco), and 100 µg/mL antibiotics (penicillin/streptomycin/amphotericin B, Biological Industries, Beit-Haemek, Israel), following ATCC guidelines. The authenticity of the TNBC cell lines was confirmed via short tandem repeat genotyping (Center for Genomic Medicine, National Cheng Kung University, Tainan, Taiwan). Radioresistant MDA-MB-231 cells, designated as 231-RR, were established as previously described. To maintain radioresistance, 231-RR cells were irradiated weekly with 2 Gy of radiation using an Elekta Axesse™ linear accelerator (Stockholm, Sweden).

### Clonogenic assay

For the clonogenic assay, 200 cells were seeded into each well of 12-well plates and allowed to attach overnight. The cells were then irradiated with 2, 4, or 8 Gy of radiation. After irradiation, the medium was replaced with fresh complete medium, and the cells were incubated for 7 days at 37°C in a humidified atmosphere with 5% CO2. Colonies were fixed with 3.7% formaldehyde and stained with 0.5% crystal violet. Colonies containing 50 or more cells were counted to determine the survival fractions.

### Purification of EVs

231-P or 231-RR cells were seeded into 6 cm dishes and incubated in complete medium containing 10% FBS until they reached 80% confluence. The cells were washed once with PBS and then cultured in 4 mL of serum-free Bio-MPM-1 medium (Biological Industries, Cat. No: 05-060-1A) for 72 hours. The culture medium was first centrifuged at 300g for 5 minutes at 4°C to remove remaining cells, followed by centrifugation at 12,000g for 30 minutes at 4°C to remove cell debris. The resulting supernatants were concentrated using 100 kDa Amicon® Ultra Centrifugal Filters (0.5 mL volume) at 14,000g until the volume was reduced to 1/150 of the initial volume. The concentrated samples were washed once with the same volume of phosphate-buffered saline (PBS) and then stored at -80°C. The particle size and concentration of the isolated EVs were measured using nanoparticle tracking analysis (NTA, NanoSight NS300, Malvern Panalytical Ltd., Malvern, UK). The structure of the EVs, including confirmation of the phospholipid bilayer, was observed using transmission electron microscopy (TEM, JEOL JEM-1400, National Cheng Kung University, Tainan, Taiwan).

### Analysis of cellular uptake of EVs

One hundred micrograms (100 µg) of purified EVs were labeled with 3,3'-Dioctadecyloxacarbocyanine perchlorate (DiO), a green fluorescent lipophilic dye (Thermo Fisher Scientific, Waltham, MA, USA), at a concentration of 10 µg/mL for 2 hours at room temperature (RT). After incubation, unbound dye was removed using a 100 kDa Amicon® Ultra Centrifugal Filter. The labeled EVs were then added to wells containing attached 231-P cells or a single-cell suspension of 231-P cells for microscopy or flow cytometry analysis, respectively. As a positive control for DiO labeling, cells were stained directly with 2 µg/mL of DiO dye at RT for 2 hours.

### Western blot analysis

EV samples or cells collected after trypsin/EDTA treatment were lysed in RIPA buffer (25 mM Tris-HCl, pH 7.6; 150 mM NaCl; 1% NP-40; 1% sodium deoxycholate; 0.1% SDS). Total protein concentration was determined using a BCA assay. Thirty micrograms (30 µg) of total protein were separated by SDS-PAGE and transferred onto PVDF membranes. The membranes were blocked with 5% skimmed milk in TBS-T buffer (50 mM Tris-Cl, pH 7.6; 150 mM NaCl; 0.05% Tween-20) for 1 hour at room temperature. Primary antibodies were incubated with the membranes overnight at 4°C. After washing with TBS-T, membranes were incubated with horseradish peroxidase-conjugated secondary antibodies for 1 hour at room temperature. After final washes with TBS-T, membranes were treated with chemiluminescence substrate, and signals were detected using a chemiluminescence imaging system (ImageQuant LAS 4000, GE HealthCare Technologies, Inc., Chicago, IL, USA). Antibody details are provided in Table S1.

### Mammosphere assay for determining self-renewal capability

A total of 2 × 10⁴ cells were suspended in mammosphere medium consisting of DMEM/F12 culture medium supplemented with 0.5% methylcellulose (Sigma-Aldrich, St. Louis, MA, USA), 0.4% BSA (Sigma-Aldrich), 1X B27 supplement (Gibco), 20 ng/mL EGF (PeproTech Asia, Rehovot, Israel), 20 ng/mL bFGF (PeproTech), 5 µg/mL insulin (Sigma-Aldrich), 4 µg/mL heparin (Sigma-Aldrich), and 1 µM hydrocortisone (Sigma-Aldrich). Cells were seeded into ultra-low attachment 6-well plates (Greiner AG, Kremsmünster, Austria) and incubated at 37°C for 7 days. Primary mammospheres were then collected using 100 µm cell strainers and dissociated with Accutase (Invitrogen, Thermo Fisher Scientific, Waltham, MA, USA) to obtain a single-cell suspension. To assess self-renewal, 5000 dissociated primary mammosphere cells were reseeded into ultra-low attachment 6-well plates to form secondary mammospheres over another 7-day incubation at 37°C. Mammosphere forming efficiency was calculated using the formula: Mammosphere forming efficiency = (number of formed mammospheres) / (initial number of seeded cells).

### Establishment of β-catenin-overexpressing 231-RR cells

The β-catenin cDNA sequence, tagged with GFP at the C-terminus, was cloned into the pLVX-EF1alpha-IRES-Puro lentiviral vector (Clontech-Takara Bio, Mountain View, CA, USA). VSV-G pseudotyped lentiviral particles were produced using Lenti-X Packaging Single Shots (Clontech-Takara Bio). Before transduction, 8 μg/mL Polybrene (Sigma-Aldrich) was added to the culture medium of MDA-MB-231-RR (231-RR) cells. The cells were then transduced with the lentiviral particles and incubated for 24 hours at 37°C with 5% CO_2_. After transduction, puromycin selection was carried out at a concentration of 2 μg/mL for 48 hours. The surviving cells were expanded for subsequent experiments, including EV purification and the detection of β-catenin delivery through EVs. The GFP tag facilitated the detection and tracking of β-catenin expression using an anti-GFP antibody, confirming successful overexpression and exosomal packaging of β-catenin.

### Analysis of gene expression profiles in breast cancer tissues

Gene expression profiles from 10 breast cancer tissue samples, collected before and after radiation treatment, were obtained from the Gene Expression Omnibus (GEO) database under the accession number GSE59733. The data were generated using the GeneChip™ Human Genome U133A 2.0 Array (Thermo Fisher Scientific Inc.). Statistical analysis was performed using an unpaired *t*-test in Prism software (version 5.0, GraphPad Software, Boston, MA, USA).

### Statistical analysis

Values of colony numbers and mammosphere formation efficiencies were expressed as mean ± standard deviation (SD). Statistical analyses were performed using GraphPad Prism 5.0. Student's *t*-test was used to compare quantitative data between two groups. One-Way ANOVA with post-hoc Tukey HSD was used for comparing data with groups more than two. A p value less than 0.05 was considered as significant difference.

## Results

### EVs released from radioresistant TNBC cells contain β-catenin

**Figure 1:**
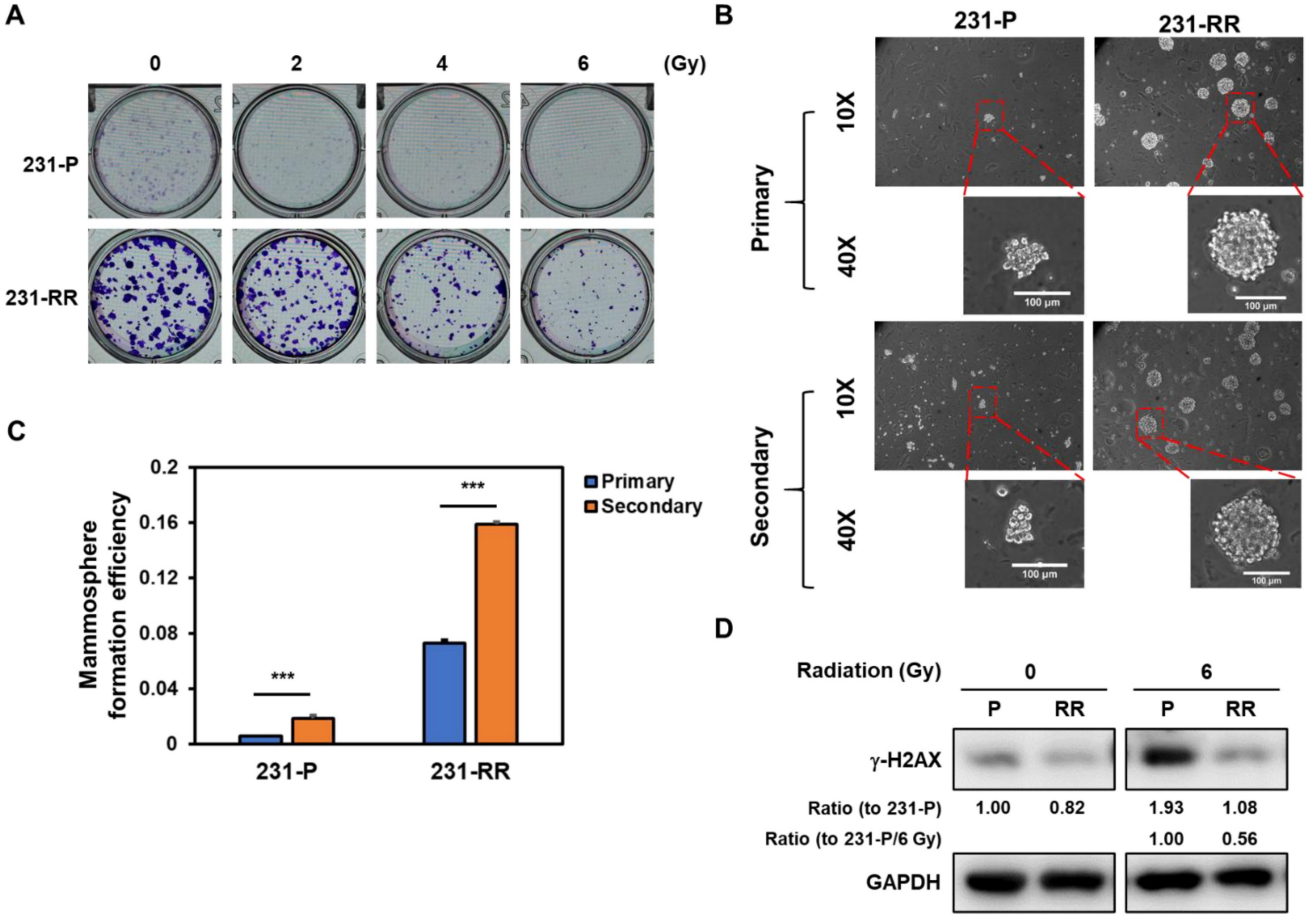
** Radioresistant MDA-MB-231 cells exhibit enhanced radioresistance, elevated CSC activity, and reduced irradiation-induced DNA damage.** (A) Clonogenic survival assay comparing parental MDA-MB-231 (231-P) and radioresistant MDA-MB-231 cells (231-RR) following exposure to increasing doses of ionizing radiation (0-6 Gy). Representative images of crystal violet-stained colonies are shown for each condition. (B, C) Representative images of primary and secondary tumorspheres are shown in (B). Each panel includes both low magnification (10× objective) and high magnification (40× objective) views. Enlarged tumorspheres correspond to regions marked with red dashed boxes in the low-magnification images. Scale bar = 100 µm in high-magnification images. Quantification of primary and secondary tumorsphere formation in 231-P and 231-RR cells are shown in (C). Data are presented as mean ± SD. ***, P < 0.001 compared to 231-P. (D) Western blot analysis of γ-H2AX expression in 231-P and 231-RR cells as a marker of DNA double-strand breaks following radiation exposure.

MDA-MB-231 cells were repeatedly irradiated with 2 Gy (totaling 32 Gy) and designated as 231-RR cells. Their radioresistant phenotype was confirmed using a clonogenic assay, where 231-RR cells formed larger colonies than parental MDA-MB-231 cells (231-P) and retained colony-forming ability at 6 Gy, unlike 231-P cells, whose colony formation was markedly inhibited (Figure 1A). Additionally, 231-RR cells showed enhanced mammosphere formation in primary and secondary assays (Figure 1B and 1C), indicative of greater cancer stem cell (CSC) capacity. Using γ-H2AX (a DNA damage marker phosphorylated at serine 139 24), we found that 231-RR cells exhibited less DNA damage than 231-P cells at 6 Gy (Figure 1D; 1.93-fold increase in 231-P vs. 1.32-fold increase in 231-RR after 6 Gy irradiation).

**Figure 2:**
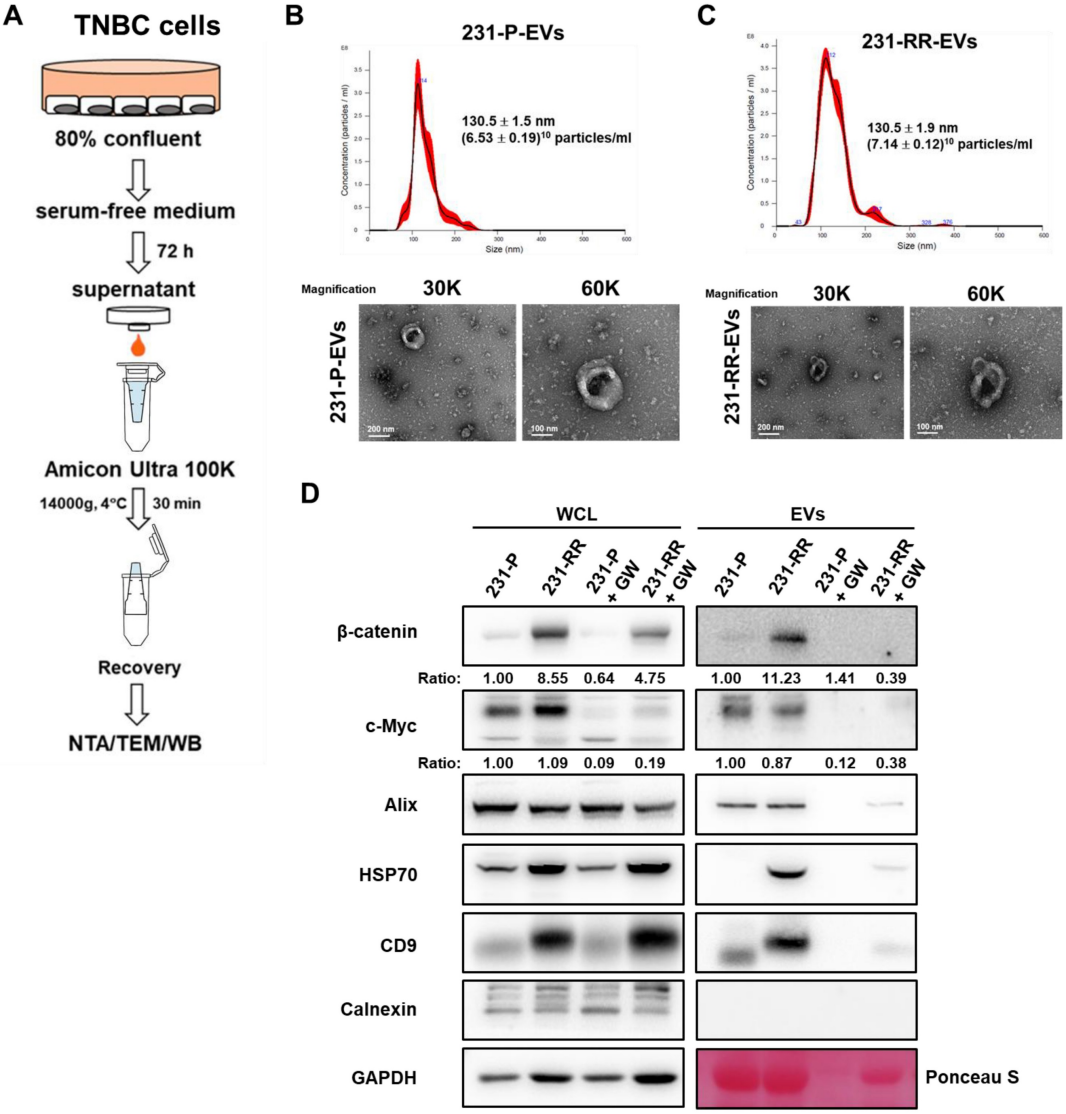
** EVs released from radioresistant MDA-MB-231 cells contain high levels of β-catenin protein**. (A) Schematic flowchart illustrating the collection and isolation EVs from TNBC cells. NTA, nanoparticle tracking analysis; TEM, transmission electron microscope; WB, western blot. (B, C) Characterization of EVs from 231-P (B) and 231-RR (C) by NTA (upper panels) and TEM (lower panels). Mean particle size and concentration (± SD) are indicated. Representative TEM images of EVs are shown at 30,000× and 60,000× magnification. Scale bars: 200 nm or 100 nm as indicated. (D)Western blot analysis of EV markers, including Alix, HSP70, and CD9, confirming positive identification of EVs. Calnexin, a negative marker for EVs, was included to verify the absence of non-EV contaminants. GAPDH was used as a control for whole cell lysates (WCL), and Ponceau S staining of the EV blot membrane served as a loading control. EVs were also examined from cells treated with 1 µM GW4869 (GW), an inhibitor of EV formation.

To compare EV release, 231-P and 231-RR cells were cultured to 80% confluence, switched to serum-free medium for 72 hours, and EVs were isolated from the culture medium by ultrafiltration. EV characterization by nanoparticle tracking analysis (NTA), transmission electron microscopy (TEM), and western blot confirmed their identity (Figure 2A). TEM revealed a typical membrane structure (lower panel of Figure 2B for 231-P-EVs and lower panel of Figure 2C for 231-RR-EVs), and NTA showed no significant difference in particle concentration between EVs from 231-P (Figure 2B, upper panel) and 231-RR cells (Figure 2C, upper panel). Western blot analysis of whole cell lysates (WCL) revealed elevated β-catenin levels in 231-RR cells (Figure 2D, WCL). In EVs, expression of exosome markers (CD9, ALIX, TSG101, and HSP70) was confirmed, with no detection of the endoplasmic reticulum marker calnexin. Notably, EVs from 231-RR cells exhibited increased β-catenin levels, but not c-Myc, compared to 231-P cells (Figure 2D, EV). Additionally, levels of HSP70 and CD9 were elevated in EVs from 231-RR cells relative to 231-P cells (Figure 2D, EV), suggesting potential differences in EV composition or biogenesis. Treatment with GW4869 (1 μM), a sphingomyelinase inhibitor that suppresses EV release 25, reduced β-catenin levels in EVs (Figure 2D, GW). These findings suggest that EVs from radioresistant TNBC cells may deliver β-catenin to neighboring cancer cells.

### EVs from radioresistant TNBC cells decrease radiation sensitivity and increase CSC activity in radiosensitive cells

**Figure 3:**
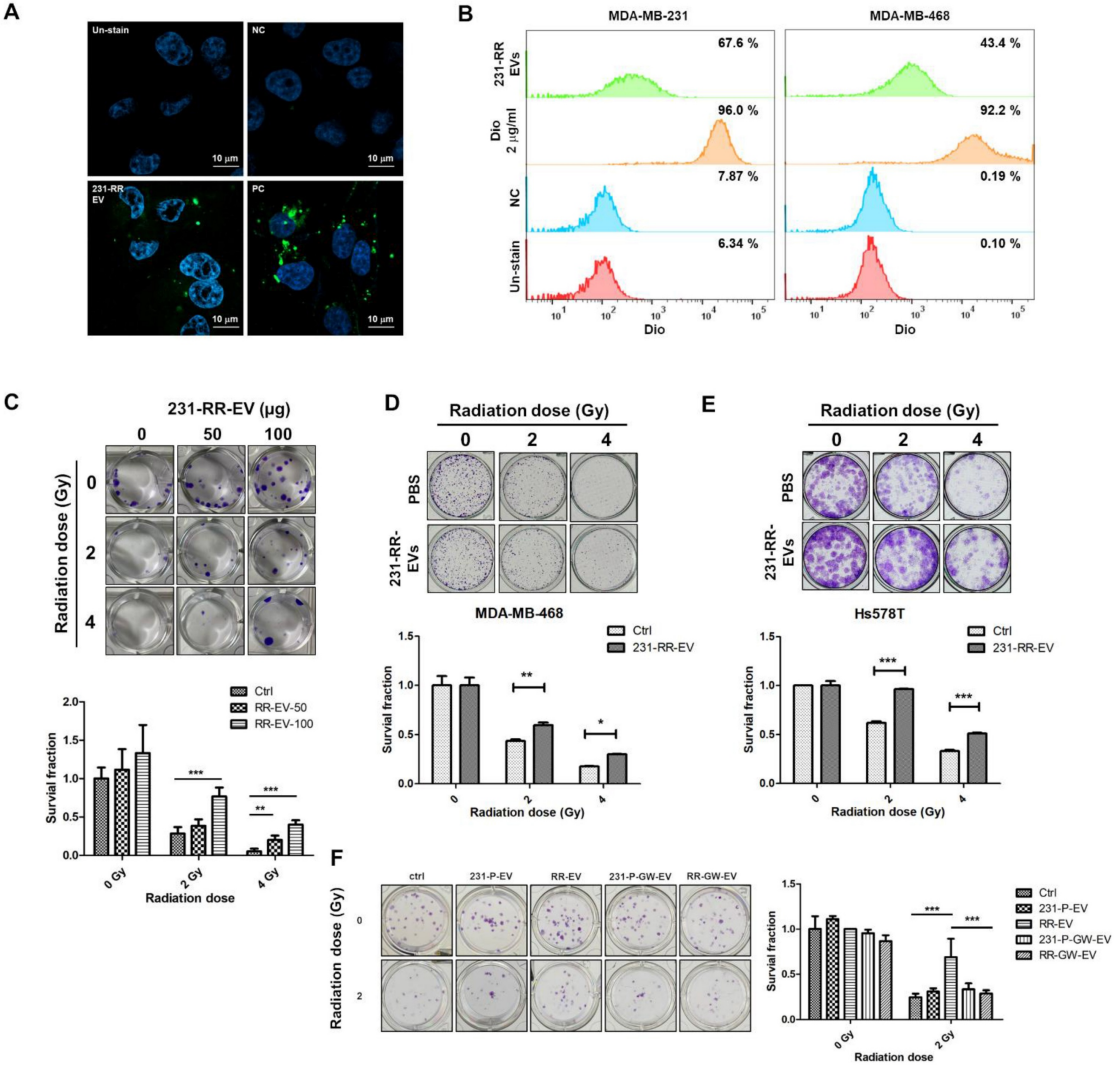
** EVs from radioresistant MDA-MB-231 cells induce a radioresistant phenotype.** (A, B) Extracellular vesicles from radioresistant MDA-MB-231 cells (231-RR-EVs) were labeled with the lipophilic green fluorescent dye DiO. Uptake of 20 µg DiO-labeled 231-RR-EVs by parental MDA-MB-231 cells was examined using confocal microscopy (A) and flow cytometry (B). MDA-MB-468 cell uptake was also analyzed by flow cytometry (B). Cells directly labeled with DiO served as a positive control (PC). PBS with the addition of 2 ug/ml Dio dye at a concentration of 2 ug/ml was also filtered through a 100KDa Amicon Ultra centrifugal filter to dead volume and collected as a negative control (NC). (C) The effect of 231-RR-EVs on the radiosensitivity of parental MDA-MB-231 cells was assessed using a clonogenic assay. MDA-MB-231 cells were treated with 50 µg or 100 µg of 231-RR-EVs for 24 hours prior to exposure to the indicated doses of radiation. (D, E) MDA-MB-468 (D) or Hs578T (E) cells were treated with 231-RR-EVs at a concentration of 100 µg followed by irradiation at the indicated doses. The radiosensitivity was determined by clonogenic assay. (F) MDA-MB-231 cells were treated with EVs from parental cells (231-P-EV), radioresistant cells (RR-EV), or from GW4869-treated cells (231-P-GW-EV, RR-GW-EV) at a concentration of 100 µg for 24 hours, followed by irradiation at 2 Gy. Colonies were visualized by crystal violet staining and counted 14 days post-seeding. Data are presented as relative colony numbers compared to the untreated control (Ctrl) without irradiation. *, P < 0.05; **, P < 0.01; ***, P < 0.001.

We next investigated the biological functions of EVs from radioresistant 231-RR cells upon treatment of TNBC cells. First, using DiO, a lipophilic fluorescent dye, to label EVs, we found that 231-RR-EVs could be taken up by 231-P cells using confocal microscopy analysis (Figure 3A). The uptake of 231-RR-EVs by 231-P and MDA-MB-468 cells was also confirmed by flow cytometric analysis (Figure 3B). The treatment of 231-P cells with 231-RR-EVs led to increased cell survival under radiation doses of 2 or 4 Gy in a dose-dependent manner (Figure 3C). These radioresistant cell-released EVs also improved cell survival of MDA-MB-468 (Figure 3D) and Hs578T (Figure 3E) under irradiations when treated at a concentration of 100 µg. The phenomenon of improved cell survival in 231-P cells under radiation by 231-RR-EVs was completely abolished in the preparation from GW4869-treated 231-RR cells (RR-GW-EVs in Figure 3F). Additionally, the treatment of 231-P cells with their own EVs did not lead to a significant increase in cell survival at 2 Gy radiation (231-P-EVs in Figure 3F).

**Figure 4:**
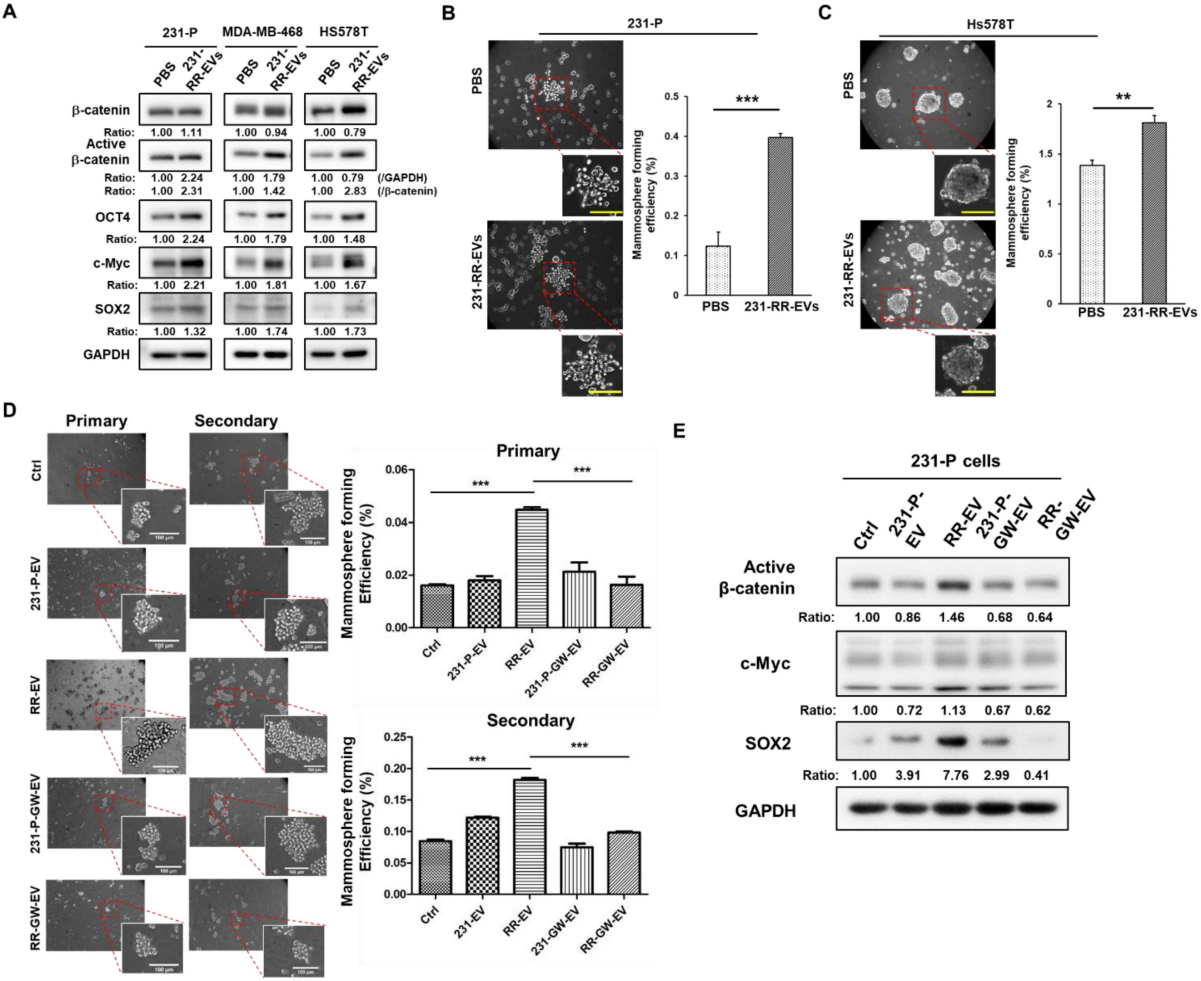
** EVs from radioresistant MDA-MB-231 cells enhance the expression of cancer stemness proteins and CSC activity.** (A) Parental MDA-MB-231 (231-P), MDA-MB-468, or Hs578T cells were treated with 100 µg of EVs derived from radioresistant MDA-MB-231 cells (231-RR-EVs) for 48 hours. The expression of cancer stemness-related proteins, including β-catenin, active β-catenin, OCT4, c-Myc, and SOX2, was analyzed by western blot. (B, C) Parental MDA-MB-231 (B) or Hs578T (C) cells were treated with 100 µg of EVs isolated from 231-P cells (231-P-EVs) for 24 hours. Representative images of mammospheres are shown (left), along with quantification of mammosphere formation efficiency (right). Each image panel includes both low magnification (20× objective) and high magnification (40× objective) images. Enlarged views correspond to areas marked with red dashed boxes in the low-magnification images. Scale bar = 100 µm in high-magnification images. **, P< 0.01; ***, P< 0.001. (D) Parental 231-P cells were treated with EVs from 231-P, 231-RR, or GW4869-treated cells (231-P-GW-EVs or RR-GW-EVs), followed by primary mammosphere formation. After 7 days, primary mammospheres were collected using 100 µm strainers, dissociated with Accutase, and single-cell suspensions were used for secondary mammosphere formation assays. Representative images include low magnification (10× objective) and high magnification (40× objective) views, with enlarged images highlighting areas marked with red dashed boxes. Scale bar = 100 µm in high-magnification images. Quantification of both primary and secondary mammosphere formation efficiency is shown. ***, P < 0.001. (E) The protein expression levels of active β-catenin, c-Myc, and SOX2 were assessed by western blot in 231-P cells treated with 100 µg EVs of 231-P, 231-RR, or GW4869 treated cells (231-P-GW-EVs or RR-GW-EVs) for 48 hours. Ratios indicated the relative expression compared to untreated control cells (ctrl).

Furthermore, treatment of TNBC cells including the parental MDA-MB-231, MDA-MB-468, and Hs578T, with 231-RR-EVs led to increased expressions of active β-catenin, as well as other cancer stemness proteins including c-Myc, OCT4, and SOX2 (Figure 4A). Enhanced CSC activity, as measured by increased mammosphere formation efficiency, was observed in both parental MDA-MB-231 (Figure 4B) and Hs578T cells (Figure 4C). To confirm the essential role of EVs, EV preparation from GW4869-treated 231-RR cells (RR-GW-EVs) was used. The increased primary and secondary mammospheres of 231-P cells by 231-RR-EVs was abolished in the treatment of RR-GW-EVs (Figure 4D). Western blot analysis further revealed that RR-GW-EVs failed to upregulate the active form of β-catenin, as well as other cancer stemness proteins of c-Myc and SOX2 in 231-P cells (Figure 4E). Together, these data demonstrate that β-catenin-containing EVs from radioresistant TNBC cells enhance CSC activity in recipient cells, potentially contributing to tumor progression.

### Inhibiting β-catenin suppresses the malignant transduction effect of EVs released from radioresistant TNBC cells

**Figure 5:**
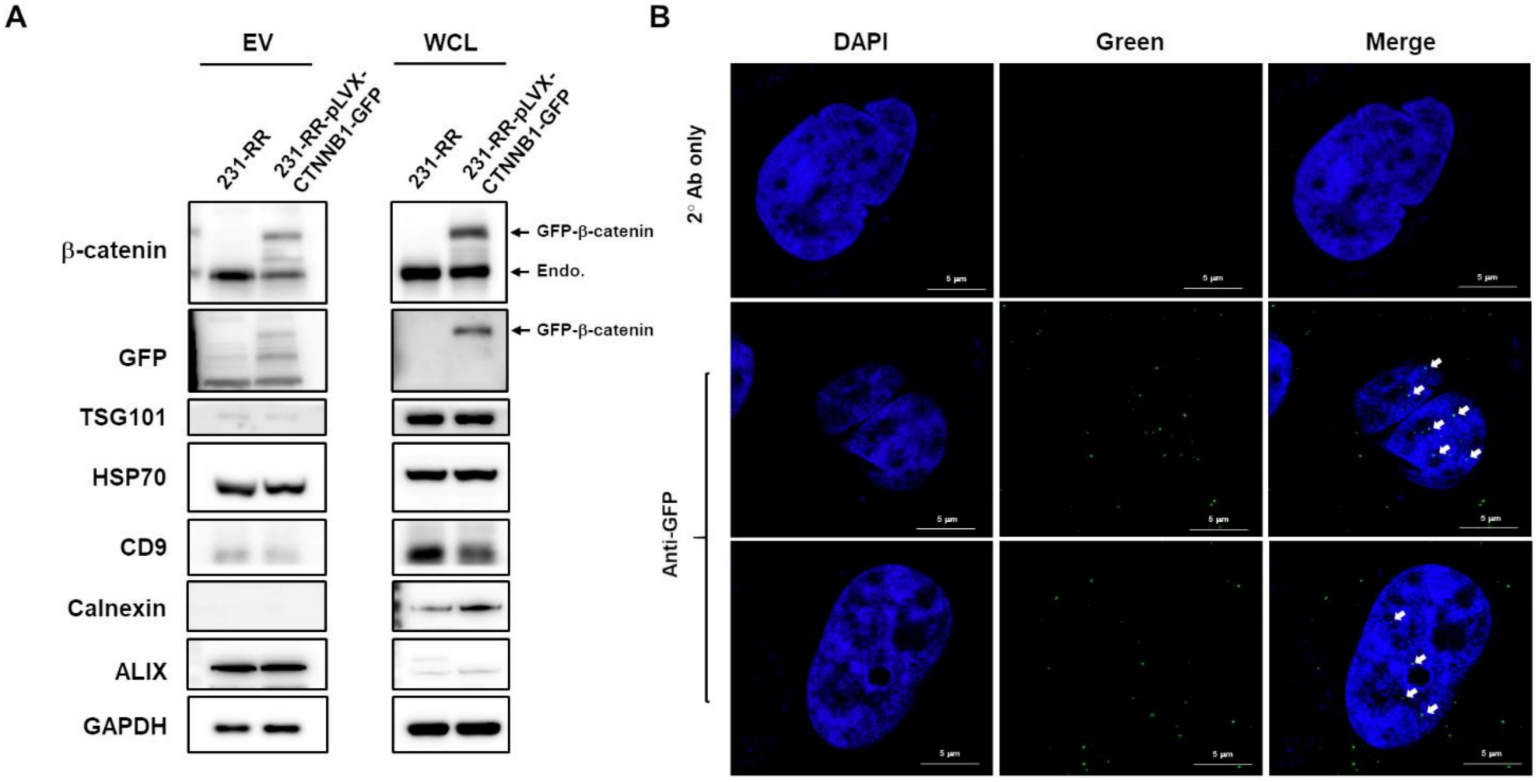
** Nuclear delivery of β-catenin by EVs released from radioresistant MDA-MB-231 cells.** (A) MDA-MB-231 cells (231-RR) were transfected with a plasmid encoding GFP-tagged β-catenin (pLVX-CTNNB1-GFP) for 24 hours, followed by puromycin selection. Whole cell lysates (WCL) and EVs released from 231-RR cells, with or without GFP-tagged β-catenin expression, were collected. Western blot analysis was performed to detect the indicated proteins. (B) Parental MDA-MB-231 cells were treated with 100 µg of EVs from 231-RR cells expressing GFP-tagged β-catenin for 2 hours. GFP expression was detected by immunocytochemical staining using an anti-GFP antibody, followed by confocal microscopy with a magnification of 630X. Arrows indicate positive GFP signals within the cell nucleus. FITC-labeled anti-mouse IgG was used as a control. Scale bars, 5 μm.

Having observed the increased β-catenin embedded in 231-RR-released EVs, we next investigated its importance in the malignant transducing effect of these EVs. We first examined the packaging and delivery of β-catenin through EVs by overexpressing GFP-tagged β-catenin in 231-RR cells using lentiviral transduction. We found that GFP-tagged β-catenin was successfully packaged into 231-RR-released EVs (Figure 5A). When these GFP-tagged β-catenin-containing EVs were added to 231-P cells, we detected the GFP-tagged β-catenin in both the cytoplasm (Figure S1) and nucleus (Figure 5B) of recipient cells by immunofluorescence staining with anti-GFP antibody.

**Figure 6:**
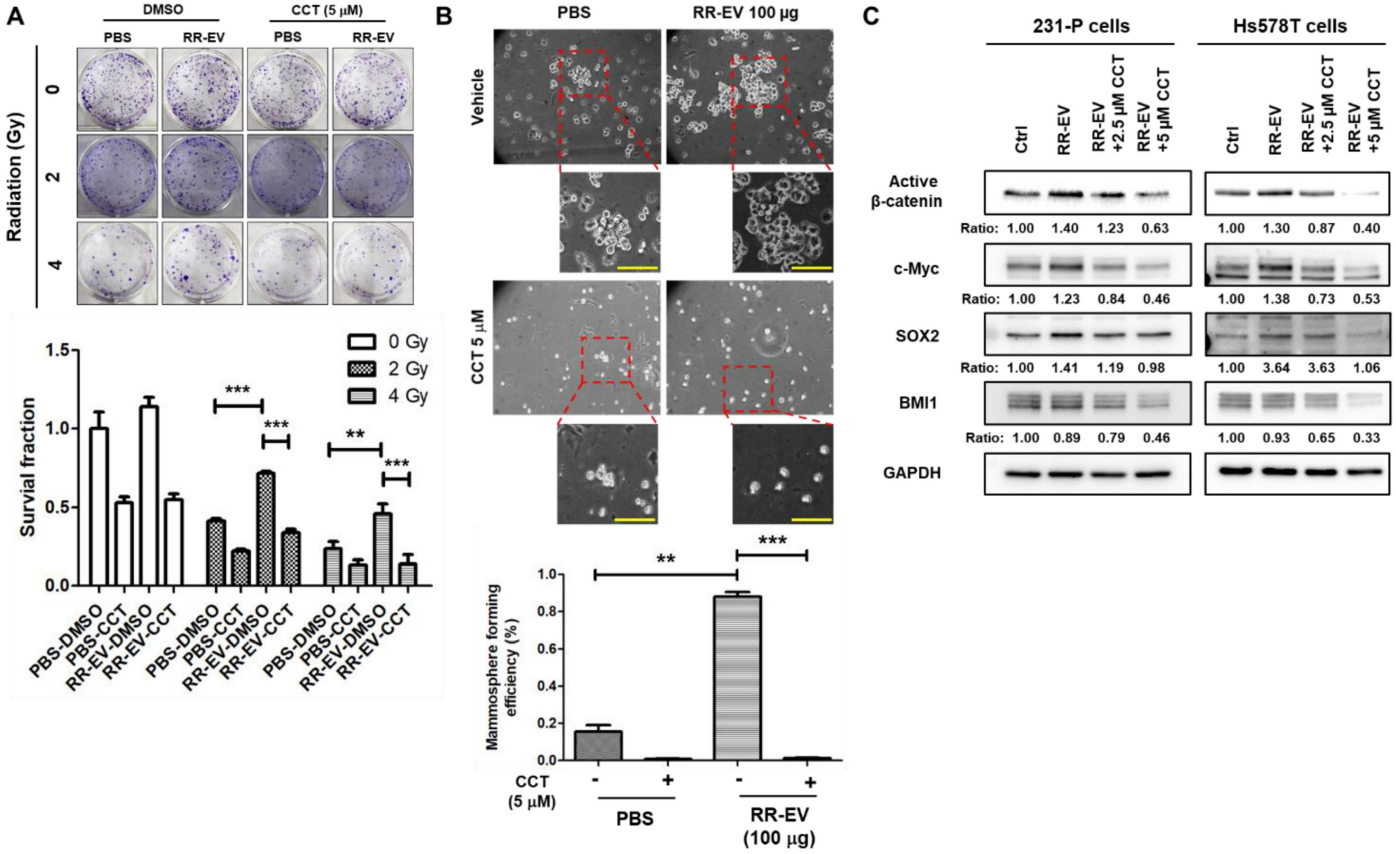
** Inhibition of β-catenin activity abolishes the effects of EVs from radioresistant cells.** Parental MDA-MB-231 cells were treated with 100 µg of EVs from radioresistant MDA-MB-231 cells (RR-EV). (A) After 24 hours of treatment, cells were irradiated with 2 or 4 Gy, and colony-forming ability was assessed by clonogenic assay with or without the β-catenin inhibitor CCT-031374 (CCT) at a concentration of 5 µM. **, P< 0.01; ***P < 0.001. (B) After 24 hours of treatment, CSC activity was measured by mammosphere formation assay with the CCT-031374 treatment (CCT, 5 µM). 0.1% DMSO was used as the vehicle control. Each image panel includes both low magnification (20× objective) and high magnification (40× objective) images. Enlarged views correspond to areas marked with red dashed boxes in the low-magnification images. Scale bar = 100 µm in high-magnification images. **, P< 0.01; ***P < 0.001. Scale bars, 100 μm. (C) After 48 hours of EV treatment in MDA-MB-231 (231-P) or Hs578T cells, with or without CCT-031374 at concentrations of 2.5 µM or 5 µM, total cellular proteins were extracted, and the expression levels of active β-catenin, BMI1, c-Myc, and SOX2 were analyzed by western blotting. GAPDH was used as a house keeping gene. Inserted numbers represent as relative levels compared to non-treated cells.

To investigate the functional role of β-catenin, we treated 231-P cells with CCT-031374, a β-catenin inhibitor 26, at a concentration of 5 μM, which maintained 75% cell survival at 72 hours (Figure S2). The inhibitor treatment abolished the 231-RR-EV-induced increase in colony formation under both 2 Gy and 4 Gy of radiation (Figure 6A). Similarly, CCT-031374 blocked the enhancement of cancer stem cell (CSC) activity induced by 231-RR-EVs (Figure 6B). Western blot analysis revealed that CCT-031374 suppressed the 231-RR-EV-induced upregulation of cancer stemness proteins, including c-Myc and SOX2 in both 231-P and Hs578T cells (Figure 6C). Although the treatment of 231-RR-EVs did not increase the BMI1 protein, the treatment of CCT-031374 reduced BMI1 levels in both 231-P and Hs578T cells (Figure 6C). These data demonstrate that radioresistant TNBC cells can transfer malignant phenotypes to neighboring cancer cells through β-catenin-containing EVs.

### Activation of β-catenin signaling in breast cancer tissues after radiotherapy

**Figure 7:**
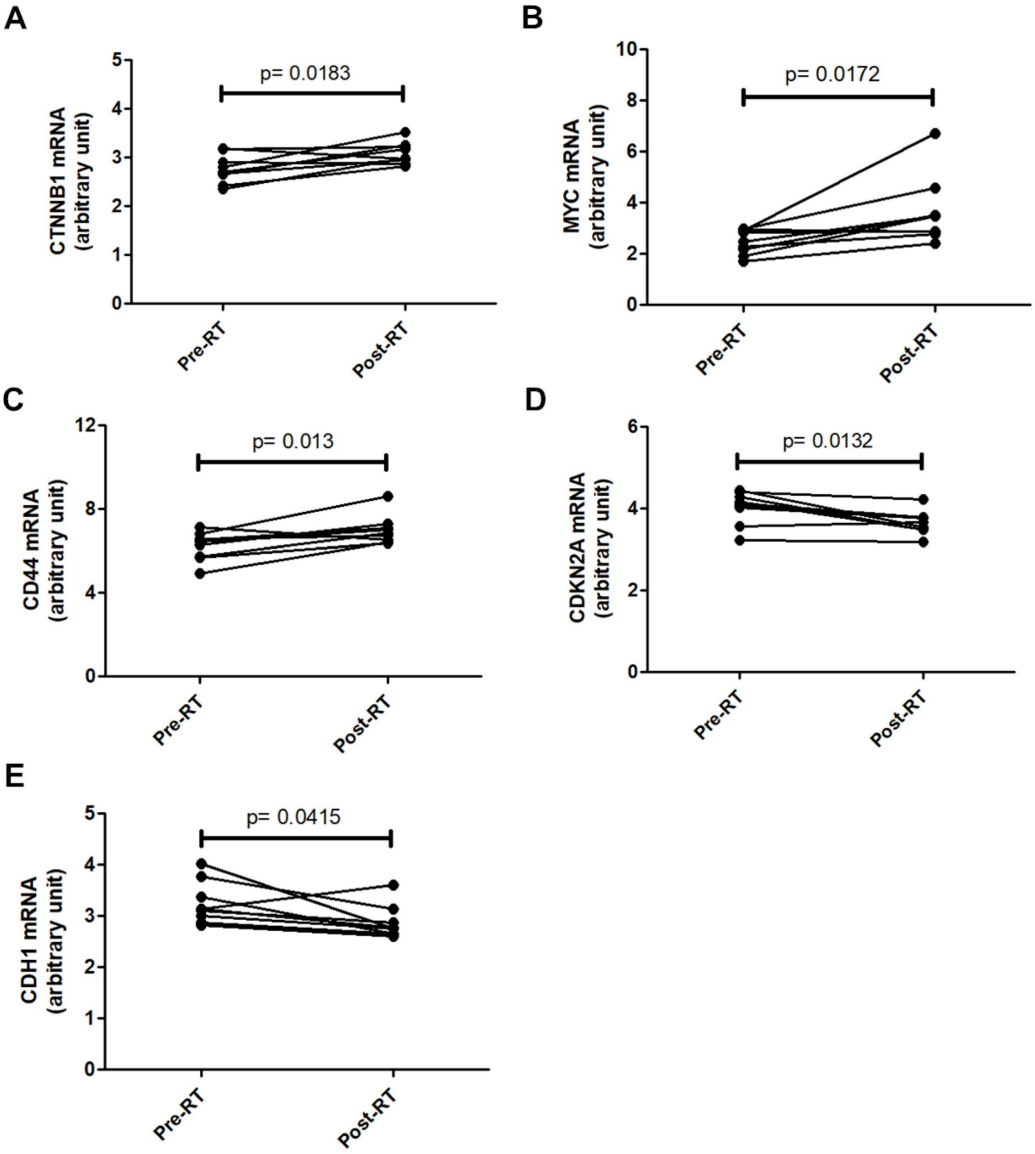
** Changes in β-catenin-related gene expression in breast cancer tissues after radio-therapy.** Gene expression data from the GSE59733 dataset were obtained from the GEO database. Expression levels of CTNNB1 (A), MYC (B), and CD44 (C), which are upregulated by β-catenin activation, were compared between breast cancer tissue samples collected before (Pre-RT) and after (Post-RT) radiotherapy. In addition, the expression levels of CDKN2A (D) and CDH1 (E), which are downregulated by β-catenin activation, were similarly compared. Statistically significant differences in expression levels are indicated with p-values.

To investigate the clinical relevance of β-catenin signaling in breast cancer radiotherapy, the GSE59733 dataset from the GEO database, including microarray data from 9 paired pre- and post-radiation tumor samples, was analyzed. Differentially expressed genes with an adjusted P value < 0.05 were analyzed using the GEO2R tool and displayed in a volcano plot (Figure S3) and listed in Table S1. Analysis of raw GSE59733 data from GEO2R revealed that β-catenin-activated genes, including *CTNNB1* (Figure 7A), *MYC* (Figure 7B), and *CD44* (Figure 7C), showed increased expression following radiotherapy. Conversely, genes negatively regulated by β-catenin, including *CDKN2A* (Figure 7D) and *CDH1* (Figure 7E), showed decreased expression following radiotherapy. These findings suggest that β-catenin pathway activation could serve as a potential predictive marker for radiotherapy outcomes in breast cancer patients.

## Discussion

Previous studies have established that cancer cells can acquire radioresistance through EV uptake. For instance, LMP1-negative nasopharyngeal carcinoma (NPC) cells gain radioresistance by taking up LMP1-bearing EVs from LMP1-overexpressing NPC cells, activating the p38 MAPK pathway 27. Similarly, in oral cancer, EVs from radioresistant cells deliver miR-503-3p to reduce BAK protein levels in recipient cells, conferring resistance to radiotherapy-induced apoptosis 21. Our study aligns with the concept of EV-mediated resistance but uncovers a novel mechanism specific to TNBC. To our knowledge, this is the first report demonstrating that β-catenin, enriched within EVs from radioresistant TNBC cells, transfers to recipient TNBC cells, reducing their radiosensitivity and enhancing CSC activity. Unlike prior findings where EV quantity or miRNA content drove resistance, our results show that while the number of EVs released by 231-RR cells did not significantly increase, the β-catenin content in EVs was markedly elevated. This β-catenin transfer resulted in heightened expression of active β-catenin and oncoproteins that positively regulates cancer stemness (c-Myc, OCT4, SOX2), amplifying malignant phenotypes such as radioresistance and CSC activity in recipient cells.

This mechanism distinguishes our findings from reports in colorectal cancer, where mutant β-catenin in EVs activates Wnt signaling without addressing radioresistance 28, or lung cancer, where EV-derived β-catenin induces epithelial-to-mesenchymal transition rather than radioresistance 29. A limitation of our study is the absence of resected TNBC tissues post-radiotherapy and reliance on the non-TNBC-specific GEO dataset GSE59733. Although this dataset demonstrates β-catenin pathway upregulation in breast cancer post-radiation, its mixed-subtype cohort limits its specificity to TNBC. Future studies with TNBC-specific patient tissues are needed to validate EV-mediated β-catenin transfer *in vivo*.

The interaction between β-catenin and SOX2 further underscores the novelty of our findings in the TNBC context. Previous research has revealed complex β-catenin-SOX2 dynamics: in MCF7 breast cancer cells, β-catenin binds SOX2 to suppress its transcriptional activity, with β-catenin knockdown increasing SOX2 function 30, while in colorectal cancer, disrupting β-catenin signaling inhibits SOX2-driven chemoresistance 31. SOX2 is a known promoter of radioresistance across cancers, including head and neck cancer 32, non-small cell lung cancer 33, cervical cancer 34, and TNBC 35. In our study, β-catenin-containing EVs from 231-RR cells enhanced radioresistance and CSC activity in recipient TNBC cells, potentially via increased SOX2 activity. This is supported by reduced SOX2 levels in 231-P cells receiving 231-RR EVs combined with CCT-031374 treatment (Figure 6C). However, the precise interplay remains unclear, and future studies inhibiting SOX2 in recipient cells could directly confirm its role in mediating β-catenin-driven effects, building on this novel observation in TNBC.

Our data demonstrate that β-catenin, transferred via EVs to recipient TNBC cells, reduces radiosensitivity and enhances CSC activity. These findings suggest that β-catenin inhibitors could serve as radiosensitizers in TNBC, potentially overcoming EV-mediated radioresistance. The concept of combining small molecule inhibitors with radiation is supported by emerging evidence in other cancer types. For example, Patel *et al.* reported that the BRAF inhibitor encorafenib and the MEK inhibitor binimetinib, when combined with stereotactic radiosurgery, improved local control of melanoma brain metastases without significant toxicity 36. Similarly, Neitzel *et al.* recently showed that ogremorphin, a small molecule inhibitor of G protein-coupled receptor 68 (GPR68), sensitizes lung and pancreatic cancer cells to ionizing radiation by increasing intracellular reactive oxygen species and inducing ferroptosis 37. Applying β-catenin inhibitors to suppress the enhanced malignant properties conferred by EV-delivered β-catenin in TNBC warrants further investigation, offering a promising strategy to improve radiotherapy outcomes in TNBC patients.

Beyond its biological role, β-catenin in EVs holds promise as a clinical biomarker, enhancing the study's significance. While EV surface molecules like glypican-1 predict pancreatic cancer progression 38, 39 or prostate-specific membrane antigen-positive EVs correlate with breast cancer survival 40, proteins in EVs like β-catenin are less studied due to detection challenges. Our findings suggest that elevated β-catenin in EVs from radioresistant TNBC cells could predict radiotherapy outcomes and recurrence risk, a possibility supported by post-radiotherapy β-catenin pathway upregulation in patient data. However, most EV-related protein biomarkers are expressed on the EV surface. However, detecting β-catenin in EVs requires advanced techniques beyond standard surface marker assays, such as mass spectrometry or western blotting 41. Given the potential low abundance of disease-specific EVs in plasma 42, sensitive single-EV analysis methods, like the NanOstirBar-EnabLed Single Particle Analysis approach 43, could overcome this bottleneck. This technique, which uses silica-coated nanorods and confocal fluorescence microscopy to detect proteins at the single-EV level, has differentiated breast cancer patients from healthy individuals using plasma samples 43, offering a pathway to translate our findings into clinical diagnostics.

## Conclusions

In summary, our study provides the first evidence that β-catenin transfer via EVs from radioresistant TNBC cells drives radioresistance and CSC activity, a mechanism distinct from prior EV-related research in other cancers. Upon uptake of these EVs, β-catenin enters the nucleus of recipient cancer cells and enhances the expression of cancer stemness factors including c-Myc, SOX2, and OCT4. This process leads to reduced radiosensitivity and increased cancer stem cell activity in the recipient cells. Importantly, blocking either EV secretion with GW4869 or β-catenin activity with CCT-031374 prevents these effects. Supporting the clinical relevance of our findings, analysis of patient data revealed activation of β-catenin signaling after radiotherapy. Our results highlight the importance of EV-mediated β-catenin transfer in radioresistance and suggest new strategies for both predicting and improving radiotherapy outcomes in TNBC patients. Future investigations into β-catenin-SOX2 interactions and single-EV detection technologies could amplify the impact of these results, positioning them as a foundation for understanding and targeting radioresistance in TNBC.

## Supplementary Material

Supplementary figures and table.

## Abbreviations

ALIX: apoptosis-linked gene 2-interacting protein X
CDH1: Cadherin 1
CDKN2A: cyclin-dependent kinase inhibitor 2A
CTNNB1: Catenin Beta 1
EVs: extracellular vesicles
MYC: MYC Proto-Oncogene, BHLH Transcription Factor
NTA: nanoparticle tracking analysis
OCT4: octamer-binding transcription factor 4
SOX2: SRY-Box Transcription Factor 2
TEM: transmission electron microscopy
TNBC: triple negative breast cancer
TSG101: Tumor susceptibility gene 101 protein
