# Supplementary material for: Radioresistant triple-negative breast cancer cells release β-catenin containing extracellular vesicles to promote cancer stem cell activity of bystanders
Source: J Cancer. 2025 Jun 23;16(9):2890–902. doi: 10.7150/jca.111555 (PMC12243990; doi:10.7150/jca.111555)
Supplement: Supplementary file 1 — Supplementary figures and table. [file jcav16p2890s1.pdf]

Supporting information

Table S1. The list of differential expressed transcripts in GSE59733 with the adjusted P-value less than 0.05.

NCBI transcript ID	Gene symbol	adj. p value[a]	log2FC[b]
NM_000389.5	CDKN1A	0.000812	1.9244
NM_004417.3	DUSP1	0.001174	2.5073
NM_001220777.2	CDKN1A	0.003963	2.9208
NM_006931.3	SLC2A3	0.005188	2.6514
NM_004417.3	DUSP1	0.013212	3.0365
NM_001202233.2	NR4A1	0.014956	2.2879
NM_004417.3	DUSP1	0.014956	3.1771
NM_002135.5	NR4A1	0.014956	2.5677
NM_001964.2	EGR1	0.014956	2.3105
NM_003407.2	ZFP36	0.014956	2.404
NM_002032.2	FTH1	0.031261	1.8593
NM_001145337.3	MDM2	0.031261	1.7767
NM_005746.3	NAMPT	0.031261	2.0363
NM_005252.3	FOS	0.039276	3.3591
NM_031476.4	CRISPLD2	0.040207	1.3426
NM_173157.3	NR4A1	0.040406	3.4081
NM_004864.2	GDF15	0.040406	2.0154
NM_002648.4	PIM1	0.043256	1.1901

[a] The adjusted P-value was calculated by the GEO2R webtool on the GEO website with the Benjamini and Hochberg method.

[b] Log2-fold change between samples of pre-radiotherapy and post-radiotherapy.

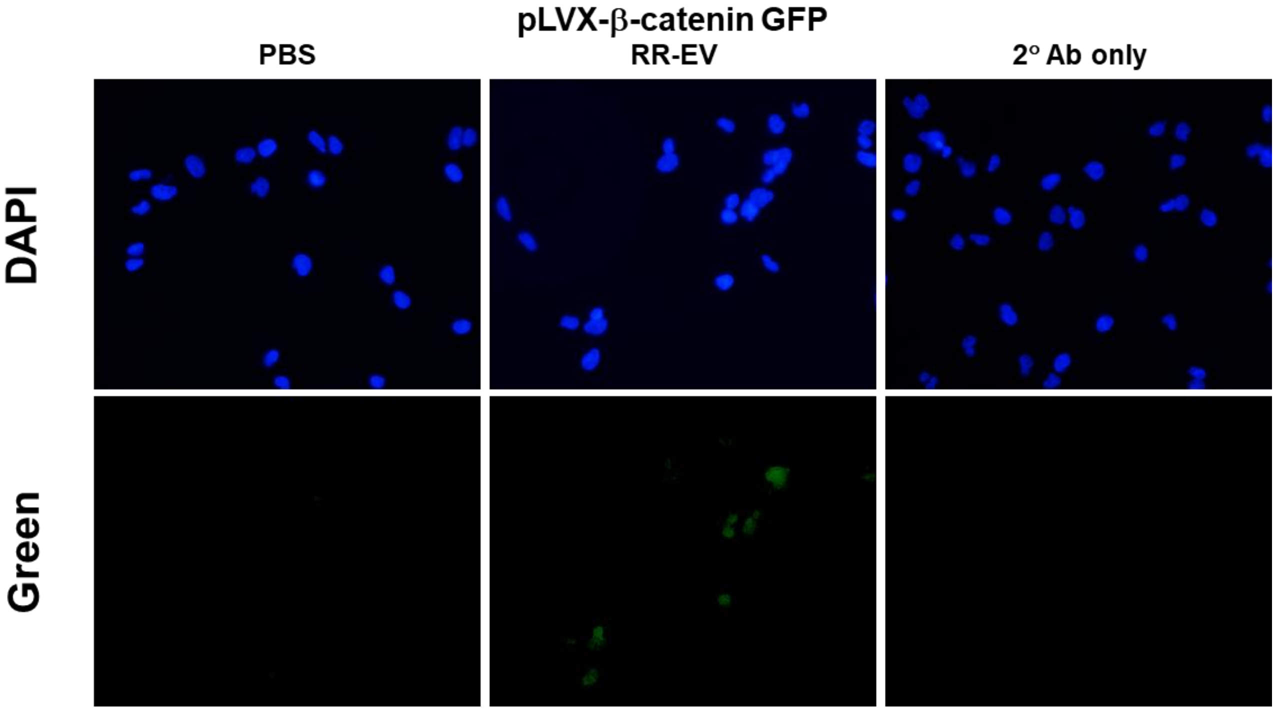

Figure S1. Examination of β-catenin delivery via EVs from radioresistant MDA-MB-231 cells. Parental MDA-MB-231 cells were treated EVs containing GFP-tagged β-catenin at 37°C for 2 hours. GFP was detected using an anti-GFP antibody, followed by staining with a FITC-conjugated secondary antibody and microscopy imaging. PBS treatment and staining with only the FITC-conjugated secondary antibody served as negative controls.

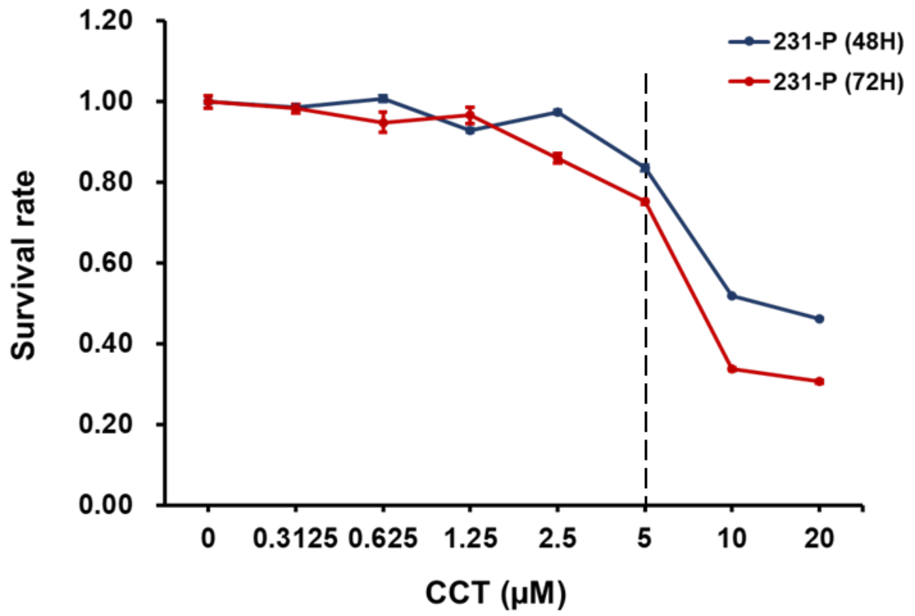

Figure S2. Cytotoxicity assay of CCT-031374 in MDA-MB-231 and radioresistant subline cells. MDA-MB-231 (231-P) and the radioresistant subline (231-RR) cells were seeded into 96-well plates and treated with the indicated concentrations of CCT-031374 for 48 or 72 hours. Cell viability was assessed using the MTT assay, and the results are presented as the percentage of surviving cells relative to the non-treated control. The dotted line represents the concentration of 5 μM.

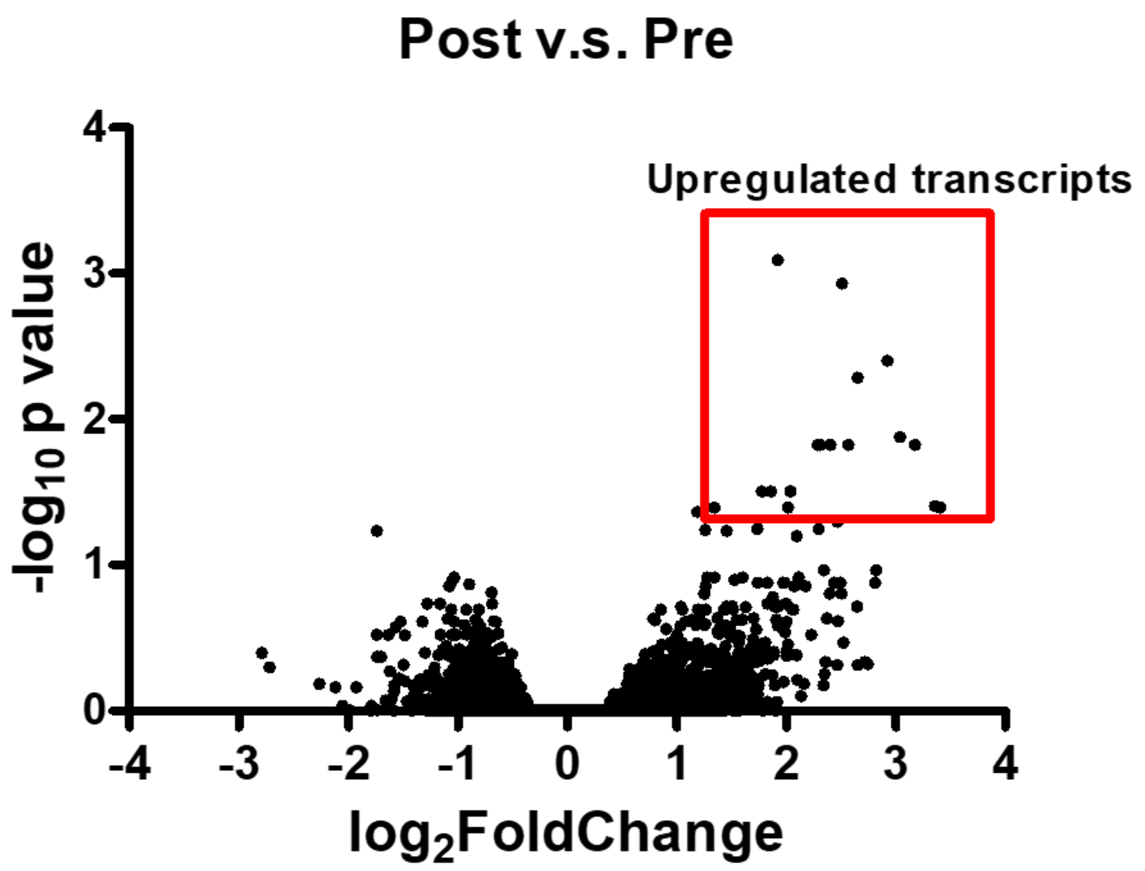

Figure S3. A volcano plot of differential expressed transcripts in GSE59733. The differential expressed transcripts were analysed by the GEO2R webtool on the GEO website, with the Benjamini and Hochberg method being applied to calculate the adjusted P-values. The Log2 fold change threshold was set as 0.3.

Whole blot images

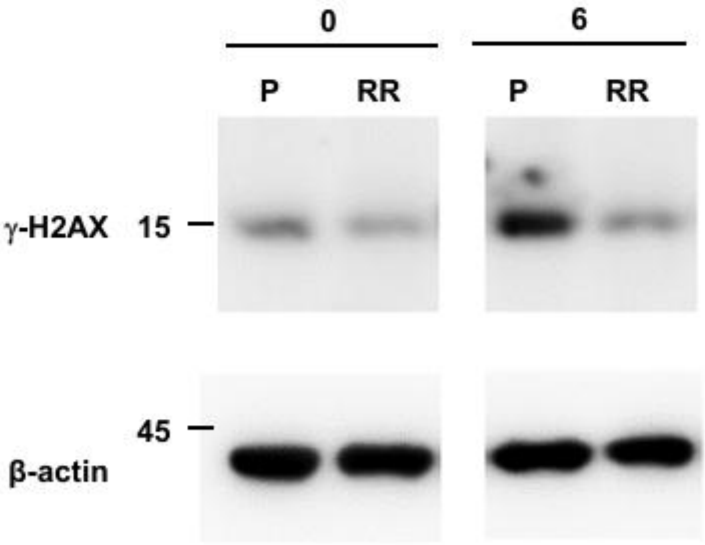

Whole blot images of Fig. 1C

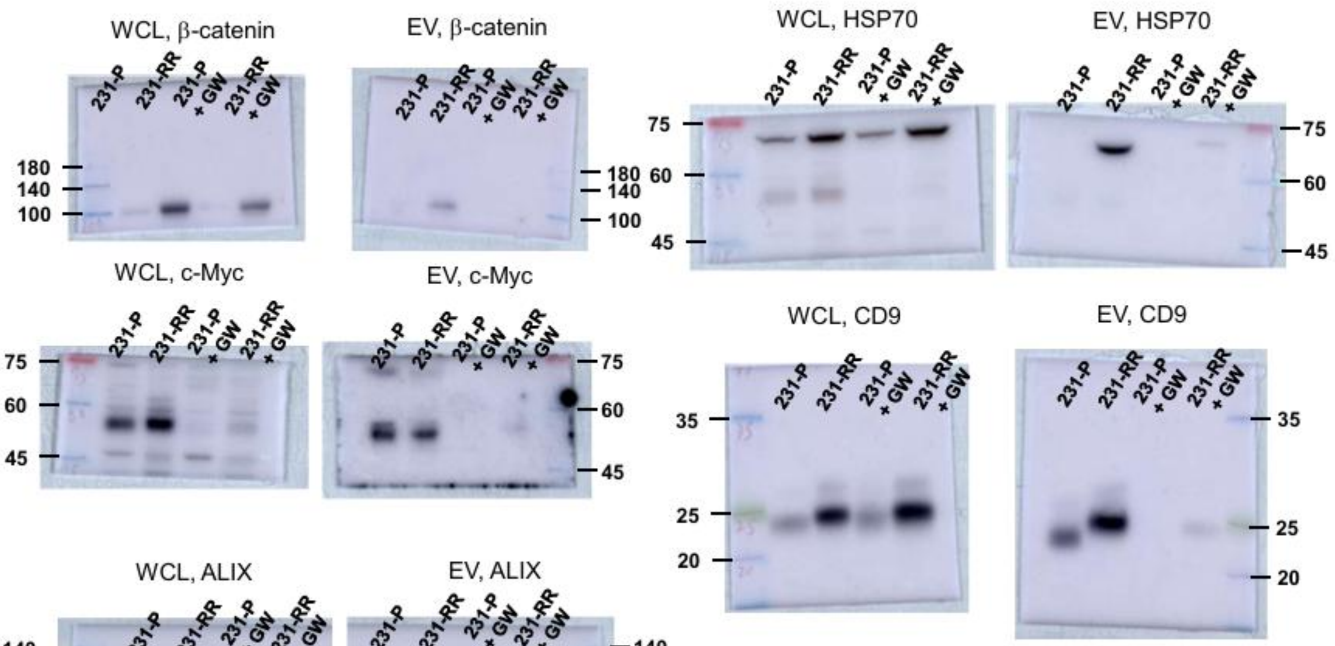

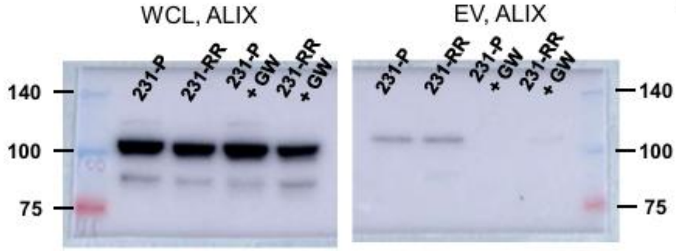

Whole blot images of Fig. 2C (part1)

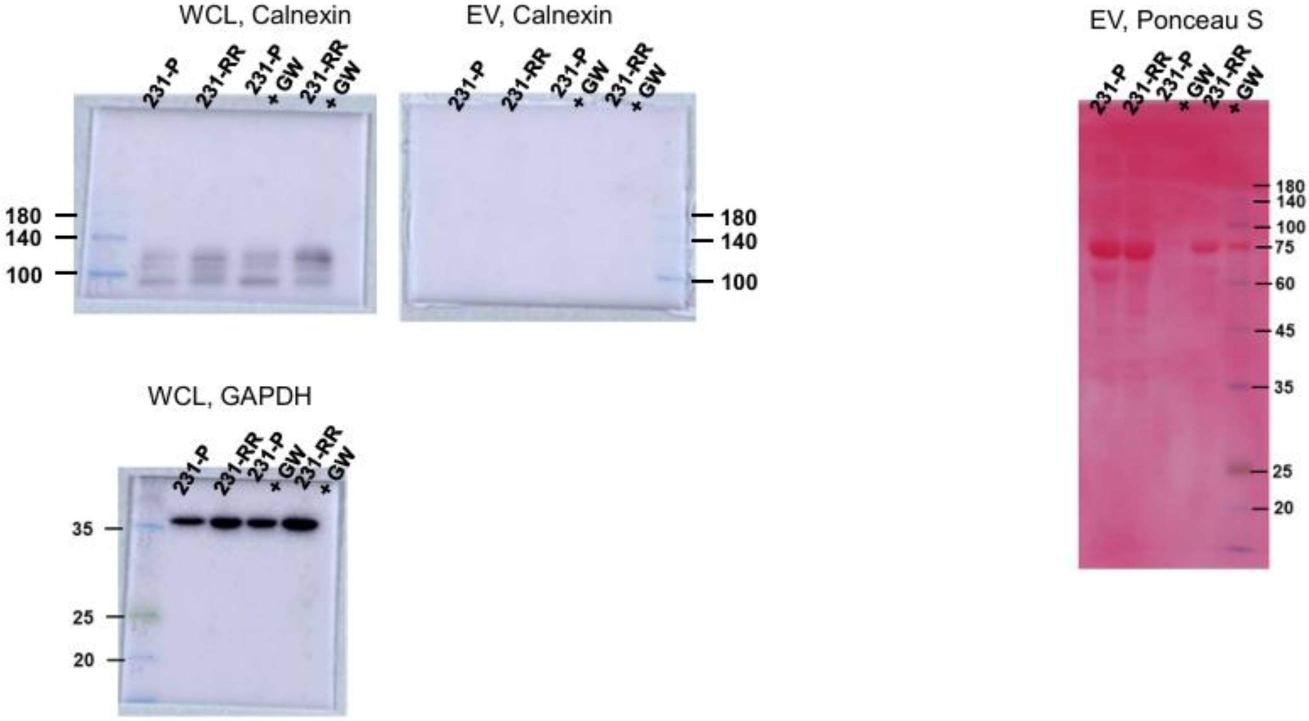

Whole blot images of Fig. 2C (part2)

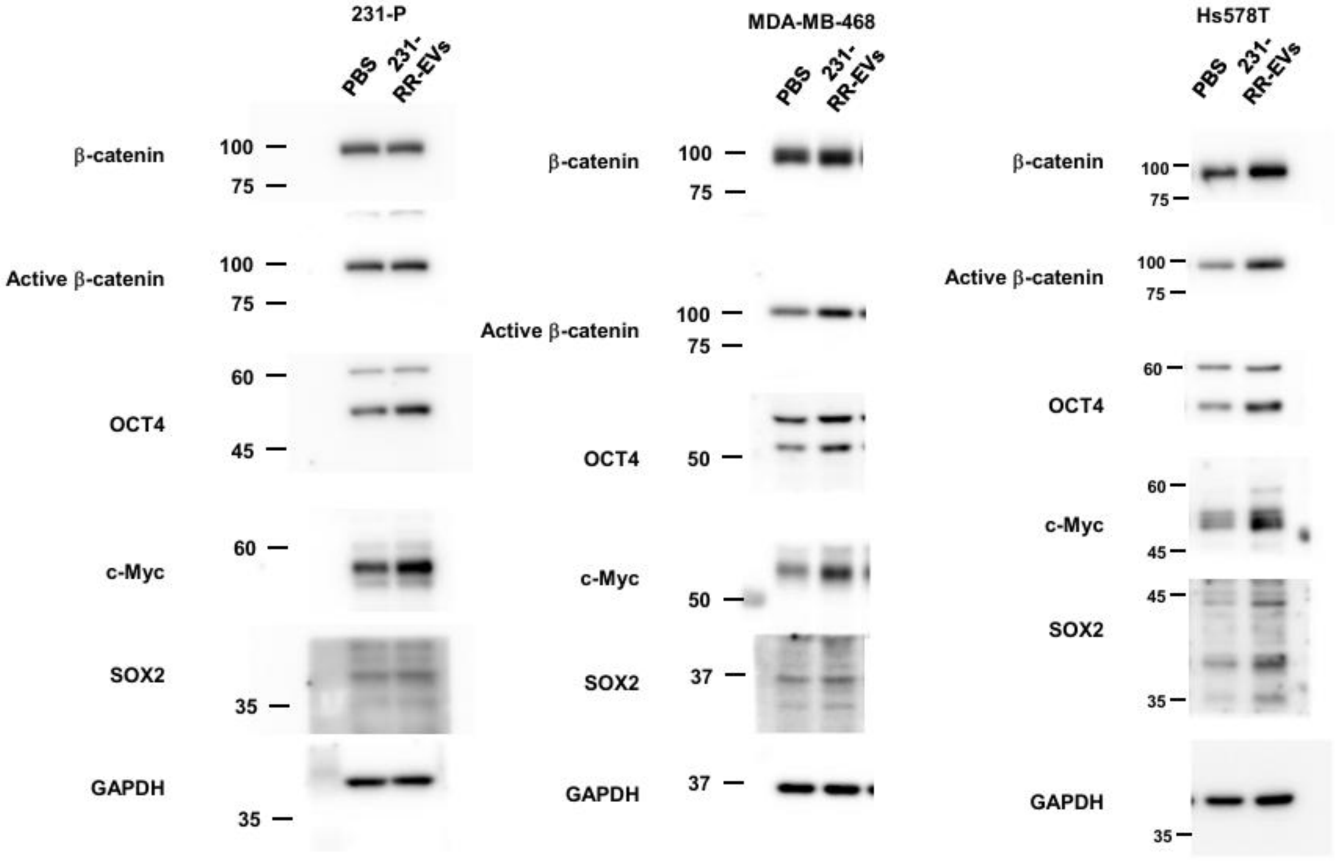

Whole blot images of Fig. 4A

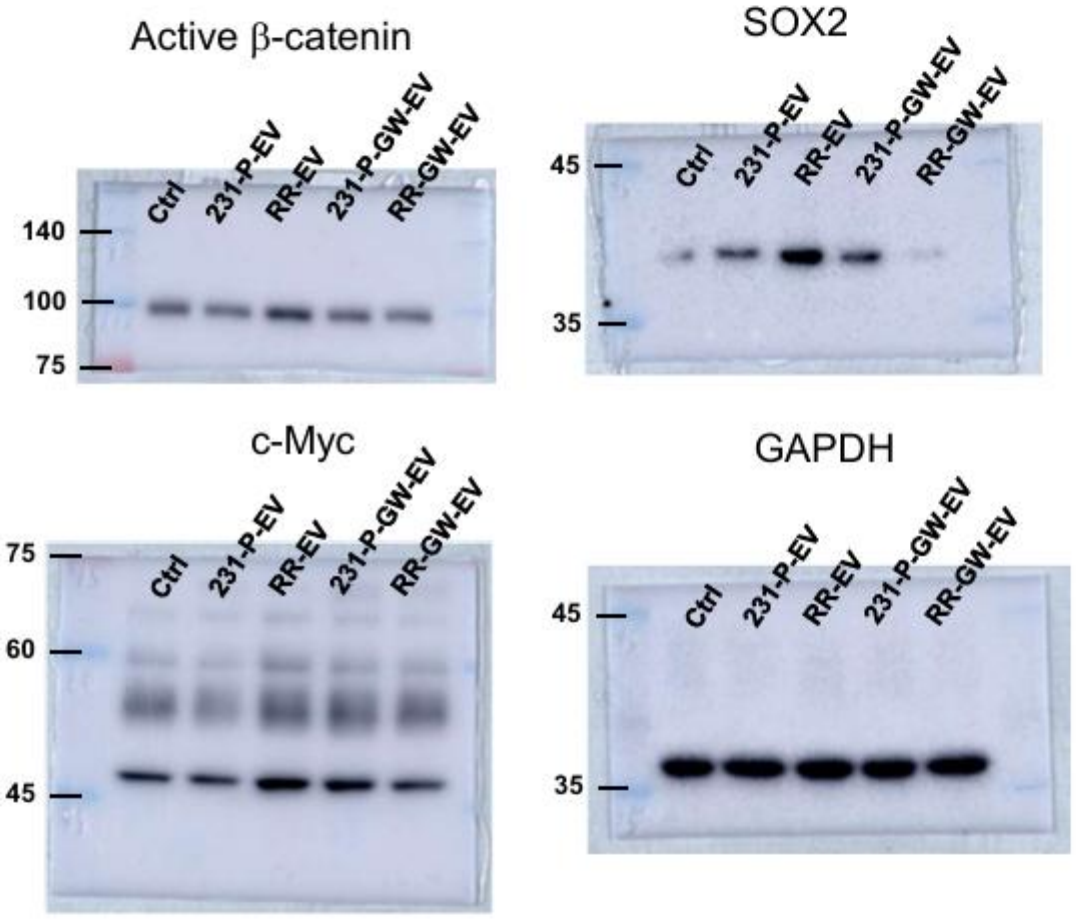

Whole blot images of Fig. 4E

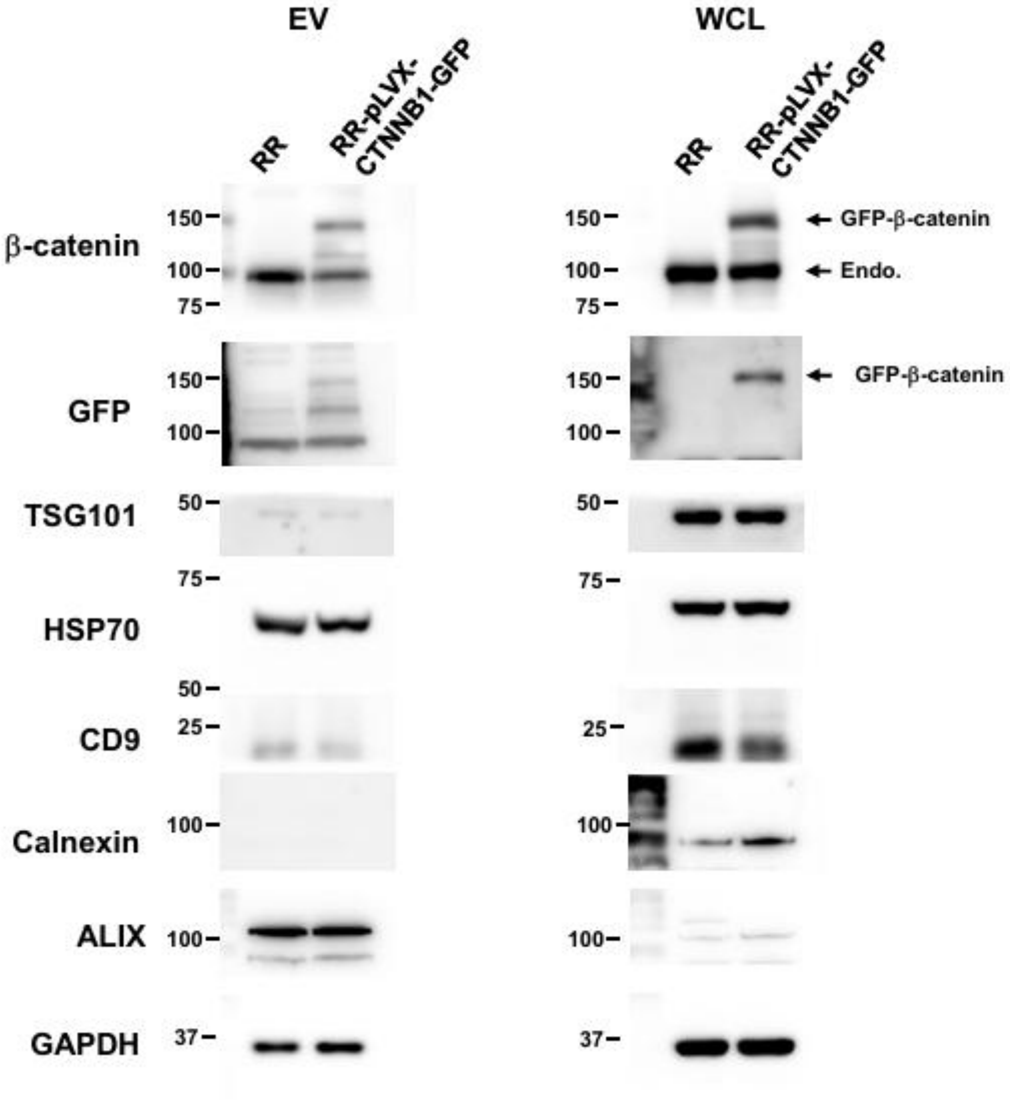

Whole lot images of Fig 5A

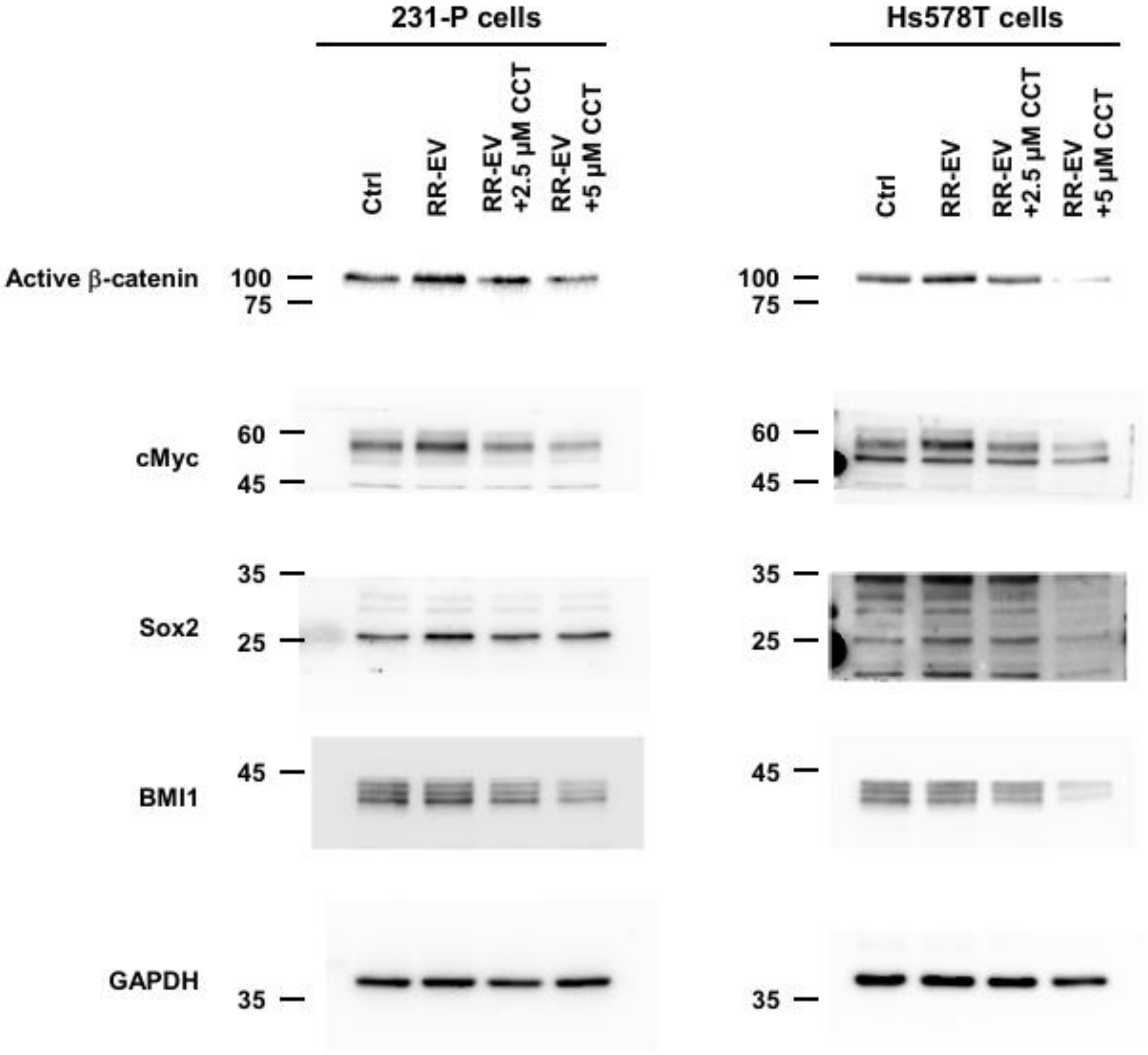

Whole blot images of Fig. 6C
